# Post-term birth as a response to environmental stress: The case of September 11, 2001

**DOI:** 10.1093/emph/eov001

**Published:** 2015-01-16

**Authors:** Claire E. Margerison-Zilko, Julia M. Goodman, Elizabeth Anderson, Alison Gemmill, Ralph A. Catalano

**Affiliations:** ^1^Department of Epidemiology and Biostatistics, Michigan State University, 909 Fee Rd. Rm. 601B, East Lansing, MI 48823, USA; ^2^School of Public Health, University of California, Berkeley, 50 University Hall #7360, Berkeley, CA 94720, USA; ^3^Department of Demography, University of California, Berkeley, 2232 Piedmont Ave, Berkeley, CA 94720, USA.

**Keywords:** critical window; parent–offspring conflict; growth; gestation; pregnancy; post-term; pre-term

## Abstract

**Background and objectives:** Despite growing interest in the role of maternal psychosocial stress as a determinant of preterm birth, no existing work has examined the relation between maternal stress and post-term birth (≥42 weeks). We hypothesize that prolonging gestation past term may represent an adaptive strategy to a suboptimal environment.

**Methodology:** We examined the relationship between exposure to the September 2001 terrorist attacks and odds of post-term birth in California. We calculated the expected odds of post-term birth among conception cohorts of singleton gestations in California between October 1996 and November 2005. We used time series analysis to test for higher than expected odds of post-term birth among the 10 cohorts exposed to the attacks of September 2001 (those conceived from December 2000 to September 2001).

**Results:** The observed odds of post-term delivery among gestations at 33–36 weeks in September 2001 were higher than statistically expected for all race/ethnic and sex groups.

**Conclusions and implications:** Our finding that odds of post-term birth were higher than expected among pregnancies exposed to the September 2001 terrorist attacks in late gestation provides initial support for the hypothesis that exposure to a psychosocial stress during pregnancy may result in prolonged gestation.

## INTRODUCTION

Post-term (≥42 weeks) birth is associated with increased risk of maternal and neonatal complications [1, 2], but we know little about the causal pathways leading to post-term delivery. Identified risk factors include increasing maternal age, obesity, nulliparity, prior post-term delivery, male fetal sex, placental sulfatase deficiency and fetal anecephaly [1, 3–5]. Despite growing interest in the role of maternal psychosocial stress as a determinant of preterm birth (<37 weeks), no existing work has examined the impacts of maternal stress on post-term birth. Empirical evidence suggests that the maternal stress response early in pregnancy may be linked to spontaneous abortion and potentially to preterm birth, and theory suggests that this may reflect a nonconscious adaptive response whereby gestations of less fit fetuses are terminated in order to conserve maternal resources for future reproduction at a more optimal time [6–8]. To our knowledge, no theoretical or empirical work has examined how post-term birth responds to maternal stress. In the remainder of this Introduction, we develop the hypothesis that post-term birth may also represent an adaptive response to maternal exposure to stress late in pregnancy. 

The exact mechanisms by which maternal psychosocial stress impacts the outcome of pregnancy remain poorly understood. Theory suggests that maternal stress hormones may serve as indicators of environmental conditions, enabling both mother and fetus to adjust adaptively to those conditions [6, 7]. The most adaptive response may change as gestation progresses. For example, although spontaneous abortion in the face of environmental stress may conserve maternal resources for future reproduction when environmental conditions are more conducive to offspring survival [9–12], this adaptive response requires that stress cues occur early enough in gestation that the fetus would not be viable if delivered.

On the other hand, stress cues later in pregnancy may not trigger parturition because this would result in a viable infant requiring care and nutrition. Birth several weeks prior to term would not conserve maternal resources or promote infant survival, especially in a suboptimal environment. Even late preterm births (33–36 completed weeks) have substantially increased risk of mortality and morbidity compared to term (37–41 completed weeks) births [13]. Further, research shows that lactation requires more daily calories than pregnancy [14], suggesting that caring for an infant in a suboptimal environment may require more maternal resources than maintaining pregnancy.

This line of reasoning leads us to the corollary that, under suboptimal environmental conditions that induce maternal stress *late* in gestation (i.e. after the fetus has reached viability), prolonging pregnancy may represent an adaptive response that protects infants until conditions improve or the infant is stronger. In particular, prolonging gestations already at term (37–41 completed weeks) may result in increased post-term birth. It is important to note, however, that risk of both maternal and infant morbidity and mortality increase after ∼41 completed weeks of gestation [1, 15, 16]. The nonconscious decisional biology that determines length of gestation may therefore tradeoff the risk to mother and baby associated with delivery in a suboptimal environment for the risk associated with later delivery in a potentially more optimal environment.

We, therefore, hypothesize that exposure to unexpected environmental stressors late in gestation will be associated with increased risk of post-term delivery. We test this hypothesis by examining the relationship between the terrorist attacks of September 2001 and the odds of post-term delivery among conception cohorts of singleton gestations in California. We chose the terrorist attacks of September 2001 as a stressor because the literature reports that the attacks resulted in severe psychological distress among Americans [17, 18] and includes several reports that low weight births as well as fetal deaths increased above expected levels after the attacks [19–24]. The increases appeared not only in New York City [19, 21, 22, 24] but also in places distant from the attacks, including California [20, 21]. Our argument implies that if these events sufficiently affected early gestation to increase fetal death, they could also extend term gestations, yielding an increase in post-term births.

We test this hypothesis separately for male and female gestations among mothers of non-Hispanic white, African American, Hispanic and Asian mothers (i.e. eight groups). We pursued stratified testing because previous research suggests that relations between environmental stressors and birth outcomes differ by sex [8, 25] and because concern over health disparities has focused attention on differences in birth outcomes by race/ethnicity.

## METHODOLOGY

### Data

The California Department of Public Health provided us gestational age (based on last menstrual period [LMP]), sex, maternal race/ethnicity and date of birth for all singletons yielded by the 110 monthly conception cohorts conceived from October 1996 to November 2005. We excluded births with improbable gestational ages as defined by Alexander *et al.* [26]. Our study population therefore included 4 376 335 singleton births. The Institutional Review Boards of the State of California and the University of California, Berkeley approved this research.

### Measures

We used these data to calculate the monthly odds of post-term birth (i.e. >41 completed weeks of gestation) among the live births yielded by the 110 monthly conception cohorts. We transformed these odds to their natural logarithms to reduce variability in variation over the cohorts and to allow us to express our results in the familiar ‘effect on odds’ metric.

Although our argument suggests that delayed parturition will affect stressed pregnancies that have reached term, it remains plausible that pregnancies stressed earlier may, upon reaching term, also benefit from lengthened gestation. We, therefore, test for higher than expected odds of post-term births for all 10 conception cohorts exposed *in utero* to the events of September 2001 (i.e. those conceived from December 2000 to September 2001).

### Analyses

Our argument implies that fetuses in the 37th–41st week of gestation during September 2001 exhibited odds of post-term birth higher than statistically expected under the counterfactual assumption that the terrorist attacks had not occurred. Researchers would typically assume that the statistically expected value under the counterfactual would equal the mean of the odds of post-term birth among a reasonable number of cohorts unexposed to the events of September 2001. This assumption could, however, lead to spurious inferences if a time series of these odds exhibited ‘autocorrelation’ including trends, cycles or the tendency to remain elevated or depressed, or to oscillate, after high or low values. The expected value of such a series is not its mean but rather the value predicted by a model that best expresses the series’ autocorrelation.

Our test identified and modeled autocorrelation in odds of post-term birth in 110 monthly cohorts conceived from October 1996 through November 2005. This approach derived the counterfactual from autocorrelation in the 10 exposed cohorts as well as from that in the 50 prior and 50 subsequent cohorts. Using fewer cohorts could have provided insufficient power to detect seasonality in which the odds in month *m* predict the odds in month *m + 12*. Using more, conversely, could have detected and specified weak autocorrelation thereby shrinking the confidence intervals of tests and making it more likely that a small movement away from the counterfactual would appear ‘significant’ [27].

We used the strategy attributed to Dickey and Fuller [28] as well as to Box and Jenkins [27] to identify and model autocorrelation in our time series. The Dickey–Fuller test detects trends and seasonal cycles as well as their combination (e.g. upwardly or downwardly trending sine waves). The Box and Jenkins routines not only model trends and cycles detected by the Dickey–Fuller test, but also identify and model the tendency of a series to remain elevated or depressed, or to oscillate, after high or low values. The Box and Jenkins approach uses ‘autoregressive’ and ‘moving average’ parameters to model such tendencies. Autoregressive parameters best describe patterns that persist for relatively long periods, while moving average parameters parsimoniously describe less persistent patterns.

As the first step in our test, we built a Box–Jenkins model of the natural logarithm of the odds of a post-term birth among non-Hispanic white, African American, Hispanic and Asian male and female fetuses in each of the 110 monthly conception cohorts. The general form of the 8 models was as follows.
(1)
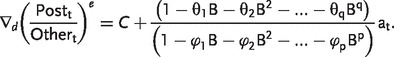



Post*_t_* is the number of infants from the cohort conceived in month *t* born at gestational age greater than 41 completed weeks. Other_t_ is the number of infants from the cohort conceived in month *t* born at gestational age less than 42 weeks. $\nabla_{d}$ is the difference operator indicating that the odds of post-term birth has been differenced at order *d* (i.e. odds at month *t* subtracted from the odds at month *t* − *d*). *C* is a constant. *θ* is the moving average, or ‘short memory,’ parameter. *ϕ* is the autoregressive, or ‘long memory,’ parameter. *a_t_* is the residual at month *t*. The ‘backshift operator,’ B^q^ or B^p^, indicates that either *θ* or *ϕ* acts on the residual ‘a’ at month *t* − *q* or *t* − *p*.

The residuals of the model have a mean of 0, constant variation and no autocorrelation. We, therefore, express them as *Z* scores that estimate their likelihood of occurrence by chance. Our argument that gestations stressed at term (i.e. 37 through 41 completed weeks) will extend to post-term suggests that the *Z* score for the cohort conceived in December 2000 will exceed 2.6 (i.e. *P* < 0.01, two-tailed test). The argument that other gestational months will prove ‘sensitive periods’ suggests that a *Z* score exceeding 2.6 will appear among the months January–September 2001.

## RESULTS

Table 1 shows the means and standard deviations for each of the eight race/ethnicity by sex groups. Table 2 shows the Box–Jenkins equations for each of the series. All the series exhibited significant autocorrelation. The odds of a post-term birth trended downward for Hispanics. All the series except those for non-Hispanic white females, African American males and Asian females also showed seasonality.

**Table 1.** Means and standard deviations of the odds of a post-term birth (≥42 weeks) in eight race/ethnicity by sex groups over 110 months

Group	Mean	Standard deviation
Non-Hispanic White males	0.0704	0.0089
Non-Hispanic White females	0.0761	0.0097
African American males	0.0712	0.0138
African American females	0.0730	0.0132
Hispanic males	0.0719	0.0091
Hispanic females	0.0780	0.0112
Asian males	0.0544	0.0084
Asian females	0.0610	0.0088

**Table 2.** Box–Jenkins models of natural logarithms of the sex- and race/ethnicity-specific odds of post-term (i.e. ≥42 weeks) as opposed to other live births among 110 monthly (i.e. October 1996–November 2005) California conception cohorts

	Males	Females
Non-Hispanic White	*Z_t_* = *C *+ 1/(1 − *ϕ*B − *ϕ*_2_B^2^) (1 − *ϕ*_3_B^12^)*a_t_*	*Z_t_* = *C* + 1/(1 − *ϕ*B) (1 − *ϕ*_2_B^5^) (1 − *ϕ*_3_B^8^)*a_t_*
African American	*Z_t_* = *C* + 1/(1 − *ϕ*B − *ϕ*_2_B^2^)*a_t_*	*Z_t_* = *C* + 1/(1 − *ϕ*B − *ϕ*_2_B^2^) (1 − *ϕ*_3_B^12^)*a_t_*
Hispanic	∇*Z_t_* = (1 − *θ*B)/(1 − *ϕ*B^12^)*a_t_*	∇*Z_t_* = (1 − *θ*B)/(1 − *ϕ*B^12^)*a_t_*
Asian	*Z_t_* = *C* + 1/(1 − *ϕ*B) (1 − *ϕ*B^12^)*a_t_*	*Z_t_* = *C* + (1 − *θ*^5^/(1 − *ϕ*B)*a_t_*

**Figure 1.**
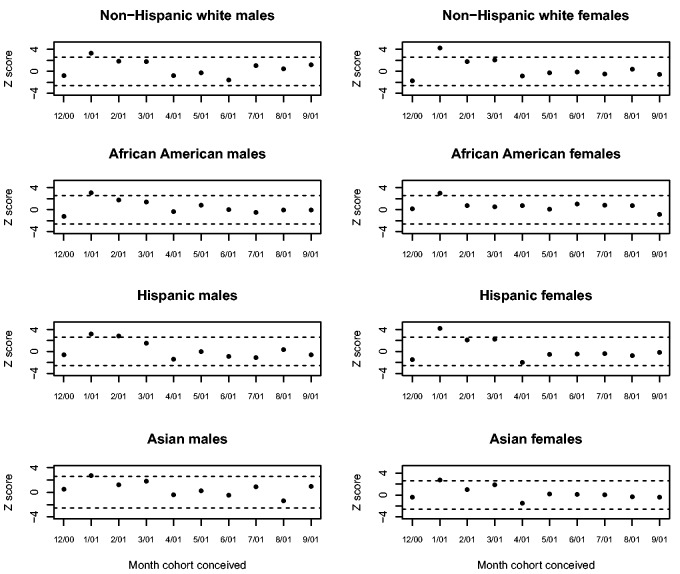
*Z*-scores for the residuals from the conception cohorts exposed to September 2001. Dotted lines show the 99% confidence interval.

Figure 1 shows the *Z* scores for the residuals from the 10 exposed race/ethnic by sex conception cohorts. The dotted lines show the 99% confidence interval. Results did not support our hypothesis that the cohorts conceived in December 2000 would exhibit scores significantly greater than 0. A ‘sensitive period’ at 33–36 weeks gestation, however, appeared for each of the eight race/ethnicity by sex group. The *Z* score for every cohort conceived in January 2001 exceeded 2.6. Hispanic males conceived in February 2001 also exhibited a value above 2.6. No residual outside the December 2000–September 2001 ‘window’ exhibited a *Z* score above 2.6. *Z* scores below −2.6 appeared for Hispanic females conceived in January and February 2002 and Hispanic males conceived in February 2002.

Table 3 shows the antilog of the nine residuals for the cohorts conceived in January 2001 and for the cohort of Hispanic males conceived in February 2001. These values estimate the ‘effect on odds’ of exposure to the events of September 2001 while in late preterm gestation. The observed odds of a post-term birth among African American males conceived in January 2001, e.g. exceeded the expected odds by 66%.

**Table 3.** Antilogs of the nine residuals in the December 2000 through September 2001 ‘window’ with *Z* scores above 2.6

	Males	Females
Non-Hispanic White	January 2001 = 1.293	January 2001 = 1.453
African American	January 2001 = 1.663	January 2001 = 1.576
Hispanic	January 2001 = 1.267; February 2001 = 1.237	January 2001 = 1.421
Asian	January 2001 = 1.417	January 2001 = 1.392

## DISCUSSION

We examined the association between exposure to the terrorist attacks of September 2001 and the odds of post-term delivery among conception cohorts of singleton births in California. Results did not support our hypothesis that cohorts conceived in December 2000 (i.e. at term in September 2001) would exhibit increased odds of post-term delivery. Cohorts conceived in January 2001 (i.e. those in the 33rd through 36th week of gestation when the attacks occurred), however, appeared much more likely to be born after 41 completed weeks.

We offer two possible explanations for our findings. The first suggests a ‘sensitive window’ in the 33rd to 36th week of pregnancy in which gestations exposed to stress are ‘programmed’ to be delivered post-term. Most gestations at 33–36 weeks would have been viable even prior to modern medical interventions and might therefore benefit from delayed parturition (potentially even more than gestations at 37–41 weeks, due to the greater fragility of infants born at 33–36 weeks). However, we know of no such mechanism that would program a gestation stressed at 33–36 weeks to be born to post-term but would not affect gestations stressed at 37–41 weeks.

Our second explanation proposes that the real stress felt by Californians was not the events occurring on September 11 itself but, rather, the subsequent fear, uncertainty, and economic collapse that intensified as the implications of the attacks became more clear. Our findings would support the argument that stress experienced at term increases post-term delivery if the stress was felt most strongly in October 2001. The current data does not permit us to discriminate between these two possible explanations for our findings.

We should also consider the duration of the population stressor. Our argument presumably applies to acute stressors for which there is reason for the decisional biology of gestation to anticipate improved conditions for delivery of an infant if gestation is prolonged by a period of days or weeks. It remains possible, however, that gestation could be prolonged even in the case of a more chronic stressor in order to deliver a hardier infant, or that the *assessment* of stress occurs repeatedly throughout late gestation, enabling the gestation to continue to extend if the stressor is prolonged. A repeated assessment of gestation could help explain our finding that gestations exposed to an initial, acute stressor (September 11) at 33–36 weeks were extended to post-term, especially given the continued stress that the population of California experienced in the weeks following September 11.

Our findings complement those suggesting that the maternal stress response early in pregnancy terminates the gestations of less fit fetuses to conserve maternal resources for future reproduction at a more optimal time [6, 8]. We propose that stress later in gestation may operate to prolong delivery until conditions improve, until the fetus is strong enough to survive in suboptimal conditions, or until the risks associated with prolonged gestation and post-term delivery outweigh the risks of delivering in a suboptimal environment.

We recognize that our argument contrasts with that which portrays adverse birth outcomes as a pathological response to stressful environments. We, however, believe that assuming gestation responds adaptively to stress [6, 7, 12] provides a more parsimonious explanation of gestational outcomes (i.e. early fetal loss, pre- and post-term delivery) than does the assumption that gestation responds pathologically to stress. While many studies attempt to identify links between stress and preterm birth, findings remain mixed [29] and clear biological mechanisms remain poorly understood [30]. Much less literature examines mechanisms leading to post-term birth.

We know of no other study that examines the relationship between maternal exposure to population stressors and post-term delivery. Strengths of our study include the use of a large (i.e. more than 3.6 million), diverse population of births over a period of more than 8 years. An important limitation of this study is that our data comprise monthly conception cohorts, so we are unable to determine exact week of gestation on 11 September 2001. An additional limitation is that measures of gestational age based on LMP can be imprecise [31], meaning that both our outcome and exposure may be subject to measurement error. Because LMP is typically assessed early in pregnancy, it is unlikely that measurement error differed substantially between pregnancies ending in late 2001 and those at other times during our study period. We also note that the terrorist attacks of September 2001 were an extreme event that may not generalize to more common sources of environmental stress. Future work should attempt to replicate these findings using less extreme population stressors.

An alternative explanation for our finding is that medical professionals and/or pregnant women became ‘distracted’ by the events of September 2001, resulting, e.g. in fewer obstetric interventions such as induction of labor or cesarean delivery or a higher likelihood of missed prenatal appointments that may have identified reasons for hastening delivery. This rival explanation would, however, also apply to gestations at term and not, as we found, only to those at late preterm.

## CONCLUSIONS AND IMPLICATIONS

Findings from this study suggest that maternal exposure to suboptimal environmental conditions during late pregnancy may be linked to prolonged gestation and post-term birth. Our findings support the argument that maternal exposure to stress later in gestation may prolong delivery until conditions improve or until the fetus is strong enough to survive in suboptimal conditions. Future work in this area should examine other types of stressors and potential differences by maternal characteristics, and should try to identify underlying biological mechanisms potentially linking stress to post-term birth.

## FUNDING

This work was supported by the Robert Wood Johnson Health and Society Scholars Program at the University of California, Berkeley and University of California, San Francisco.

**Conflict of interest**: None declared.
